# Methamphetamine self-administration causes neuronal dysfunction in rat medial prefrontal cortex in a sex-specific and withdrawal time-dependent manner

**DOI:** 10.3389/fphar.2025.1527795

**Published:** 2025-02-14

**Authors:** Lihua Chen, Tabita Kreko-Pierce, Stefanie L. Cassoday, Lena Al-Harthi, Xiu-Ti Hu

**Affiliations:** Department of Microbial Pathogens and Immunity, RUSH University Medical Center, Chicago, IL, United States

**Keywords:** meth-SA, mPFC, neuronal excitability, VGCCs, sex differences, short-term and long-term-withdrawal, electrophysiology

## Abstract

Methamphetamine (Meth) is one of the most widely used illicit drugs worldwide, exerting potent psychostimulant effects that fuels its highly addictive nature. Chronic Meth use is associated with severe cognitive impairments, particularly in executive functions, decision-making, and working memory, which persist long even after cessation of Meth use. These cognitive deficits are associated with dysfunction of glutamatergic pyramidal neurons in the medial prefrontal cortex (mPFC), which regulates addiction and cognition. Both human and animal studies highlight Meth-induced mPFC dysfunction that contributes to compulsive behaviors and relapse. Emerging evidence also highlights significant sex differences in Meth use disorder (MUD). Previous studies suggest that Meth-induced behavioral and neuronal dysfunctions are different between males and females, but the cellular and molecular mechanism are not fully understood. Using behavioral and electrophysiological approaches (whole-cell patching), this study determined certain sex differences in neuronal dysfunction in the mPFC of rats that self-administered Meth (Meth-SA) followed by a short (2–5 d) or long-term (≥30 d) withdrawal. We found that both male and female rats self-administered methamphetamine in a similar pattern; however, the resulting hypoactivity, hyperactivity, and calcium dysregulation in mPFC neurons differed between the sexes. Such sex-specific neuronal dysfunction was associated with, and depended on, short or long-term withdrawal, respectively. By understanding these sex-specific behavioral/neuronal differences following different Meth withdrawal period, our novel findings demonstrate the role of sex as a biological variable in Meth-use and relapse, and reveal the effects of drug-using environment on mPFC neuronal dysfunction during withdrawal, providing insights for gender-specific treatment strategies.

## Introduction

According to the United Nations Office on Drugs and Crime (UNODC, 2021), amphetamine-type stimulants, including methamphetamine (Meth), are the second most widely used illicit drugs worldwide. Meth acts as a potent psychostimulant, significantly impacting the central nervous system by increasing the release and blocking the reuptake of dopamine, norepinephrine, and serotonin (Hyman and Malenka, 2001; Hart et al., 2012; Ferrucci et al., 2019; Miller et al., 2021). These effects drive Meth’s highly addictive nature, leading to a vicious cycle of compulsive drug use, dependence, withdrawal, and relapse. Chronic Meth use is associated with severe cognitive impairments, particularly in areas such as working memory, executive function, and decision-making, which can persist even after 11 months of abstinence (Hart et al., 2012; Panenka et al., 2013; Bernheim et al., 2016). Despite these widespread and harmful effects, there are currently no effective treatments for Meth use disorder (MUD).

Addictive memories are processed through a network involving several key brain regions, including the mPFC, ventral tegmental area (VTA), and nucleus accumbens (NAc), each contributing to the storage and retrieval of drug-related memories (Everitt and Robbins, 2015; Flores-Dourojeanni et al., 2021). While the precise hub of this network remains unclear, the mPFC is widely considered to play a central role in orchestrating the integration of internal and external information relevant to addictive memory processing (Goldstein and Volkow, 2011; Euston et al., 2012). The mPFC is essential for regulating executive functions such as task flexibility, goal-directed behavior, working memory, and problem-solving, with deficits in mPFC function resulting in a loss of inhibitory control and driving compulsive drug-seeking behaviors (Goldstein and Volkow, 2011; Euston et al., 2012; Wayman et al., 2015; 2016). Specifically, the mPFC has been identified as a critical region for the retrieval of Meth-associated memories (Euston et al., 2012; Anastasiades and Carter, 2021). Both clinical and preclinical research suggests that the mPFC’s activation during Meth-related memory retrieval is positively correlated with drug craving intensity, highlighting its vital role in behaviors related to addiction and relapse (Otis et al., 2018; Zhao et al., 2021; Zhang et al., 2022). Chronic MUD is strongly associated with cognitive decline, particularly in the mPFC. Both human and animal studies link these cognitive deficits in episodic and working memory to reduced mPFC activity often referred to as hypofrontality (Scott et al., 2007; Goldstein and Volkow, 2011; Armenta-Resendiz et al., 2022).

Clinical and preclinical studies have identified key sex differences in the progression and severity of MUD. Women typically begin using Meth at a younger age and escalate more rapidly to regular use compared to men (Brecht et al., 2004; Roth and Carroll, 2004; Reichel et al., 2012; Bernheim et al., 2016). They also display stronger dependence on the drug and are more likely to experience severe consequences, including higher relapse rates and more pronounced neuropsychiatric symptoms such as anxiety, depression, and psychosis (Roth and Carroll, 2004; Cox et al., 2013; 2016; Hartwell et al., 2016; Daiwile et al., 2022). These sex differences extend to animal models, where female rodents exhibit greater Meth-seeking behaviors and enhanced locomotor activity, while males show more significant alterations in dopamine signaling (Roth and Carroll, 2004; Becker et al., 2012; Reichel et al., 2012). Despite this, little is known about the underlying cellular and molecular mechanisms that drive these sex differences in the mPFC and whether distinct sex-specific mechanisms are employed during short-term *versus* long-term withdrawal from Meth.

In the current study we focus on understanding sex differences in Meth use by examining functional changes in the mPFC pyramidal neurons of male and female rats. Specifically, we investigate sex-specific variations in drug-taking behavior and the activity of pyramidal neurons in the mPFC following both short-term (2–5 days) and long-term (30 days) withdrawal from Meth-self administration (Meth-SA). The goal of this study to uncover how these neuronal and behavioral differences contribute to addiction and relapse, highlighting the critical role of sex as a biological variable in understanding Meth use and its effects on brain function.

## Materials and methods

### Animals

Adult male and female F344 rats of the same age were purchased from Charles River Laboratories (Wilmington, MA, United States) and were housed and cared for at Rush University Comparative Research Center which is fully accredited by the Association for Assessment and Accreditation of Laboratory Animal and licensed by the United States Department of Agriculture. The animal study was approved by Rush University Medical Center IACUC. The study was conducted in accordance with the local legislation and institutional requirements. Animals were group-housed (2 rats per cage) in polycarbonate cages at room temperature on a 12-h light/dark cycle, with food and water available *ad libitum*. All animal use and experimental procedures in these studies were conducted in accordance with NIH, USDA and institutional guidelines, and approved by the Institutional Animal Care and Use Committee at Rush University Medical Center. All studies were performed on 4–5-month-old male and female rats (equivalent to17∼18-year-old humans) (Sengupta, 2013) with the body weight of 200–220g and 160–180 g, respectively. A total of 94 rats were used for this study, comprising 49 females and 45 males. For the short-term withdrawal study, 29 female rats (Meth-SA: n = 15; SAL-Yoked: n = 14) and 28 male rats (Meth-SA: n = 15; SAL-Yoked: n = 13) were utilized. For the long-term withdrawal study, 20 female rats (Meth-SA: n = 11; SAL-Yoked: n = 9) and 17 male rats (Meth-SA: n = 9; SAL-Yoked: n = 8) was utilized. Following the behavioral tests, these animals were sacrificed for subsequent electrophysiological studies. For the neuronal firing study following short-term withdrawal nine female rats (SAL-Yoked: n = 4 rats, 11 cells; Meth-SA: n = 5 rats, 18 cells) and 10 male rats (SAL-Yoked: n = 5 rats, 11 cells, Meth-SA: n = 5 rats, 15 cells) were utilized. For neuronal firing study following long-term withdrawal 11 female rats (SAL-Yoked: n = 5 rats, 10 cells, Meth-SA: n = 6 rats, 10 cells) and eight male rats (SAL-Yoked: n = 4 rats, 13 cells; Meth-SA: n = 4 rats, 12 cells) were used. For the Ca^2+^ spike study following short-term withdrawal 10 female rats (SAL-Yoked: n = 5 rats, 10 cells, Meth-SA: n = 5 rats, 11 cells) and 14 male rats (SAL-Yoked: n = 7 rats, 13 cells; Meth-SA: n = 7 rats, 11 cells) were utilized. For the Ca^2+^ spike study following long-term withdrawal 12 female rats (SAL-Yoked: n = 5 rats, eight cells; Meth-SA: n = 7 rats, 11 cells) and 11 male rats (SAL-Yoked: n = 6 rats, eight cells; Meth-SA: five rats, 10 cells) were used. Some animals were utilized for both neuronal firing and Ca^2^⁺ spike analyses. This overlap means that the total number of animals used is smaller than the sum of animals reported for each individual experiment.

### Meth self-administration and drug-seeking

**FIGURE 1:**
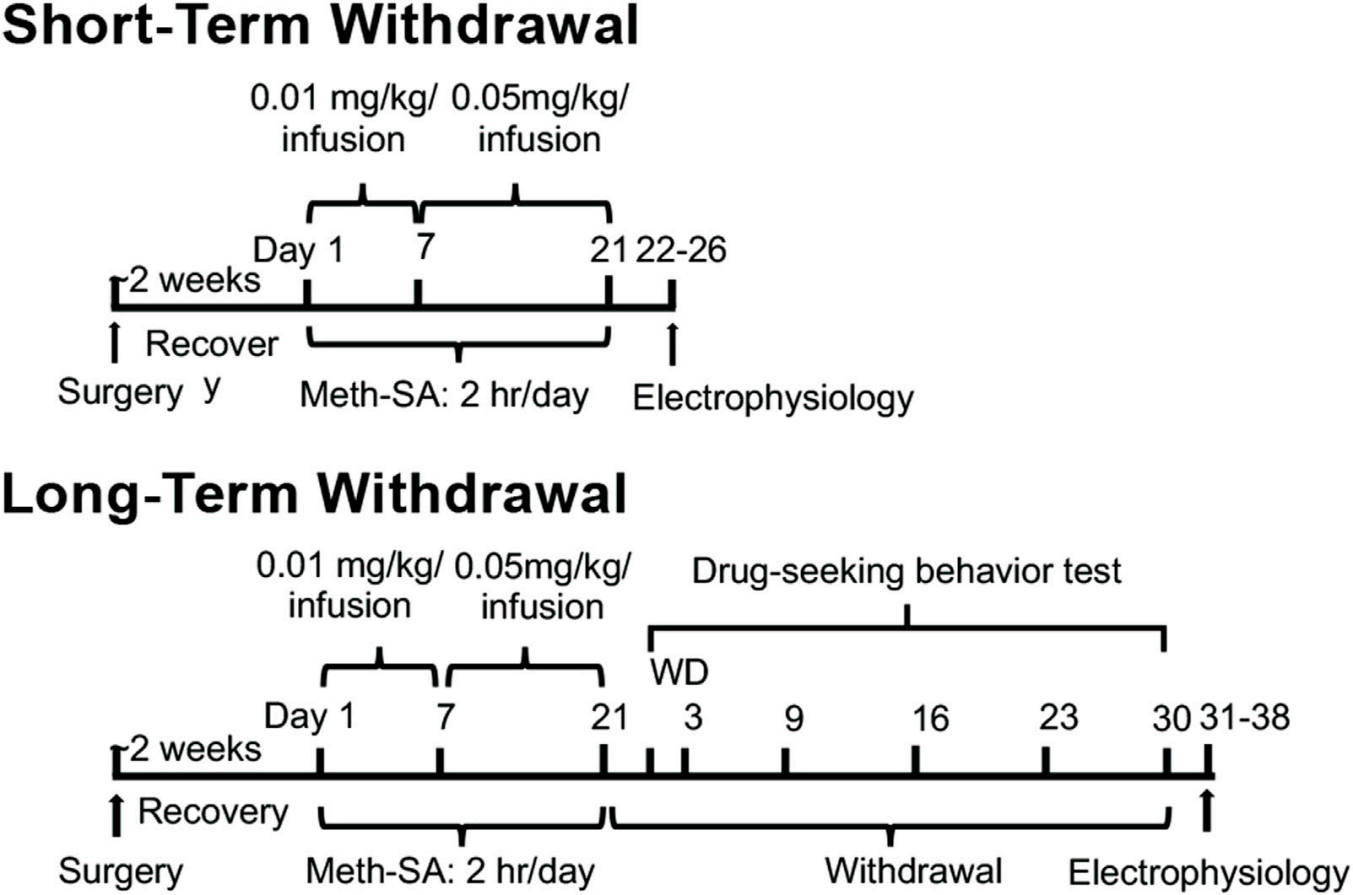
Experimental timeline and design. Male and female rats were given approximately 2 weeks to recover from surgery, during which they were implanted with intravenous (i.v.) catheters for SA assays. Following recovery, the rats were trained for Meth-SA at a dose of 0.01 mg/kg/0.1 mL per infusion for 2 h per day over 7 days. This was then increased to 0.05 mg/kg/0.1 mL per infusion for 2 h per day for an additional 14 days, for a total training period of 21 days. After completing the Meth-SA phase, the rats were sacrificed for patch-clamp analysis of neuronal activity. Neuronal activity was assessed either 2–5 days post-final infusion (short-term withdrawal) or 30 days post-final infusion (long-term withdrawal). During the long-term withdrawal phase, the animals were also evaluated for drug-seeking behavior on withdrawal days 2, 3, 9, 16, 23, and 30.

The timeline for experimental procedures is shown in Figure 1. Intravenous (i.v.) catheterization surgery was performed as previously described (Graves et al., 2015; Wayman et al., 2015; 2016) on 4–5-month-old male and female rats. Briefly, rats were deeply anesthetized with isoflurane and implanted with catheters constructed of silastic tubing (Instech Laboratories, Inc., Plymouth Meeting, PA, United States) in the right jugular vein. Rats were given approximately 2 weeks to recover from surgery prior to self-administration (SA) training period. During SA, the rats were placed in operant chambers (Med-Associates, St. Albans, VT) and trained to self-administer Meth by performing nose-pokes (NP). Each operant chamber (working area: 11.63″ x 9.25″ x 10.75″, base: 21″ x 13.75″ x 0.5″) had a stainless-steel grid floor and was equipped with a house light, stimulus lights positioned above each NP hole, and a speaker, and the entire setup was enclosed in a sound-attenuating cubicle (interior dimensions: 21.81″ x 21.88″ x 15.94”; exterior dimensions: 25.94″ x 24″ x 19″) (Med Associates Inc., Fairfax, VT). Rats were connected to a tether leash with a counterbalance weight, allowing them to move freely within the chamber. Each successful NP in the active hole triggered the activation of a light and tone stimulus, along with a 6-s infusion of Meth delivered via a variable speed syringe pump (flow rate: 1.064 mL/min; speed: 3.33 RPM; Med Associates Inc., Fairfax, VT). NPs in the inactive hole produced no response. The SA training sessions were conducted for 2 h/day, with each NP resulting in an infusion of Meth at a dose of 0.01 mg/kg/0.1 mL infusion. Rats were trained for 7 days to self-administer METH (Meth-SA) and then switched to 0.05 mg/kg/0.1 mL infusion 2 h/d for 14d (total 21d) (Figure 1). Saline (SAL)-Yoked rats were used as controls. After a short-term withdrawal (2-5d), or a long-term withdrawal (30d), from the last SA infusion, rats were prepared for electrophysiological assessment of neuronal activity. During the 30-day withdrawal period, the rats were placed in the SA boxes for a 2h drug-seeking behavior test on the withdrawal day (WD) 2, 3, 9, 16, 23, and 30. To assess drug-seeking behavior during withdrawal, we implemented a cue-reactivity protocol. During this phase, rats were returned to the operant chambers for 2 h/day; however, the methamphetamine or saline syringes were removed, ensuring no drug delivery occurred. To maintain consistency with prior SA sessions, the rats remained tethered to the infusion system. Active NPs continued to elicit light and tone cues previously associated with Meth infusions, serving as conditioned stimuli. Throughout the training and withdrawal periods, animals were placed in the operant chambers for 2 h each day. After each session, they were returned to their housing cages with unrestricted access to food and water.

### Slice preparation

Rats were deeply anesthetized with isoflurane followed by transcardiac perfusion with ice-cold oxygenated (95% O_2_ and 5% CO_2_) cutting solution (in mM: 248 sucrose, 2.9 KCl, 2 MgSO_4_, 1.25 NaH_2_PO_4_, 26 NaHCO_3_, 0.1CaCl_2_, 10 glucose, three kynurenic acid, one ascorbic acid; pH = 7.4–7.45) and rapid dissection of the cortex. The cortex was immediately place in and ice-cold oxygenated cutting solution and left to recover for 2 min. Coronal brain slices (300 µm) containing mPFC were cut using a vibratome (Leica Biosystems, Buffalo Grove, IL, United States) and transferred to a recovery chamber filled with oxygenated artificial cerebrospinal fluid (aCSF) containing (in mM): 125 NaCl, 2.5 KCl, 25 NaHCO_3_, 1.25 NaH_2_PO_4_, 1 MgCl_2_, 2 CaCl_2_, 15 glucose. Slices were incubated at RT for 1 h in aCSF and kept under continuous oxygenation until transferred to the recording chamber.

### Electrophysiology

All electrophysiological recordings were made at room temperature. During recordings, slices were superfused with oxygenated aCSF at a flow rate of ∼2 mL/mL using a recirculating pump. Pyramidal neurons in mPFC were visualized with DIC contrast microscopy using a ×40 water-immersion objective on a Nikon Eclipse E600FN microscope (Nikon Instruments Inc., Melville, NY, United States). Electrodes were pulled from borosilicate glass (4–6 MΩ) on a Fleming/Brown micropipette puller (Sutter Instruments, Novato, CA, United States) and were filled with internal solution (pH = 7.3–7.35; 280–285 mOsm); for evoked action potentials (in mM: 120 K-gluconate, 10 HEPES, 0.1 EGTA, 20 KCl, 2 MgCl_2_, 3 Na_2_ATP, and 0.3 NaGTP); or voltage-gated Ca^2+^ spikes (in mM: 140 Cs-gluconate, 10 HEPES, 2 MgCl_2_, 3 Na_2_ATP and 0.3 NaGTP), in mPFC pyramidal neurons. To measure mPFC excitability, action potentials (APs) were evoked by depolarizing current pulses (0–300 pA) for 500 msec at 25 pA intervals. Criteria for data analysis of APs in these studies included 1) a stable RMP more hyperpolarized than −60 mV, and 2) a rheobase (minimal depolarizing current)-evoked, initial action potential with amplitude greater than 60 mV. To isolate and assess VGCC activity, 0.5 µM tetrodotoxin (TTX), 20 mM tetraethylammonium (TEA), 2.5 mM kynurenic acid and 100 µM picrotoxin (PTX) was added to the perfusing bath solution to block voltage gated Na^+^ channels, K^+^ channels, NMDA/AMPA receptors and GABA_A_R respectively. For Ca^2^⁺ current recordings, cells were maintained at a holding potential of −67 mV Ca^2+^ plateau potentials (indicating Ca^2+^ influx *via* voltage-gated Ca^2+^ channels, VGCCs) were elicited by a minimal depolarizing current (rheobase) with a 40 ms duration. Criteria for data analysis included 1) consistent rheobase-evoked Ca^2+^ spikes, and 2) perfusion with aCSF containing ion channel blockers/receptor antagonists for at least 10 min.

### Statistics

All data were analyzed using Student’s t-test, two-way ANOVA or two-way repeated measures (rm) ANOVA followed by Sidak’s *post hoc* test. Initially, three-way rmANOVA was performed when three independent variables were present, and in all cases, there was no significant three-way interaction. Therefore, data analysis is displayed using the two-way rmANOVA statistics. Two-way rmANOVA was used to compare the Meth-SA daily behavior and current-spike # relationships. Two-way ANOVA was used to compare Ca^2+^ spike area and duration. Student’s t-test was used to analyze Meth total intake. The number of animals and neurons per group assessed in this study was determined from power analysis in combination with empirical data from our previously published studies. Outliers were excluded as defined as ≥2× the standard deviation from the mean or if the experimental criteria were not met (as stated in electrophysiology section) (Khodr et al., 2016). Data are presented as Mean ± SEM. *p* < 0.05 was considered as statistically significant.

## Results

Clinical and animal studies have demonstrated notable sex differences in Meth use and withdrawal, with males and females displaying distinct behavioral and neurophysiological responses during both short-term and long-term phases. To explore these sex-specific mechanisms further, we conducted a Meth-SA experiment using 4–5-month-old male and female rats. Following this, we assessed the function of pyramidal neurons in the mPFC after both short-term and long-term withdrawal periods. The full experimental timeline is summarized in Figure 1.

### Both male and female rats self-administered meth (Meth-SA)

**FIGURE 2:**
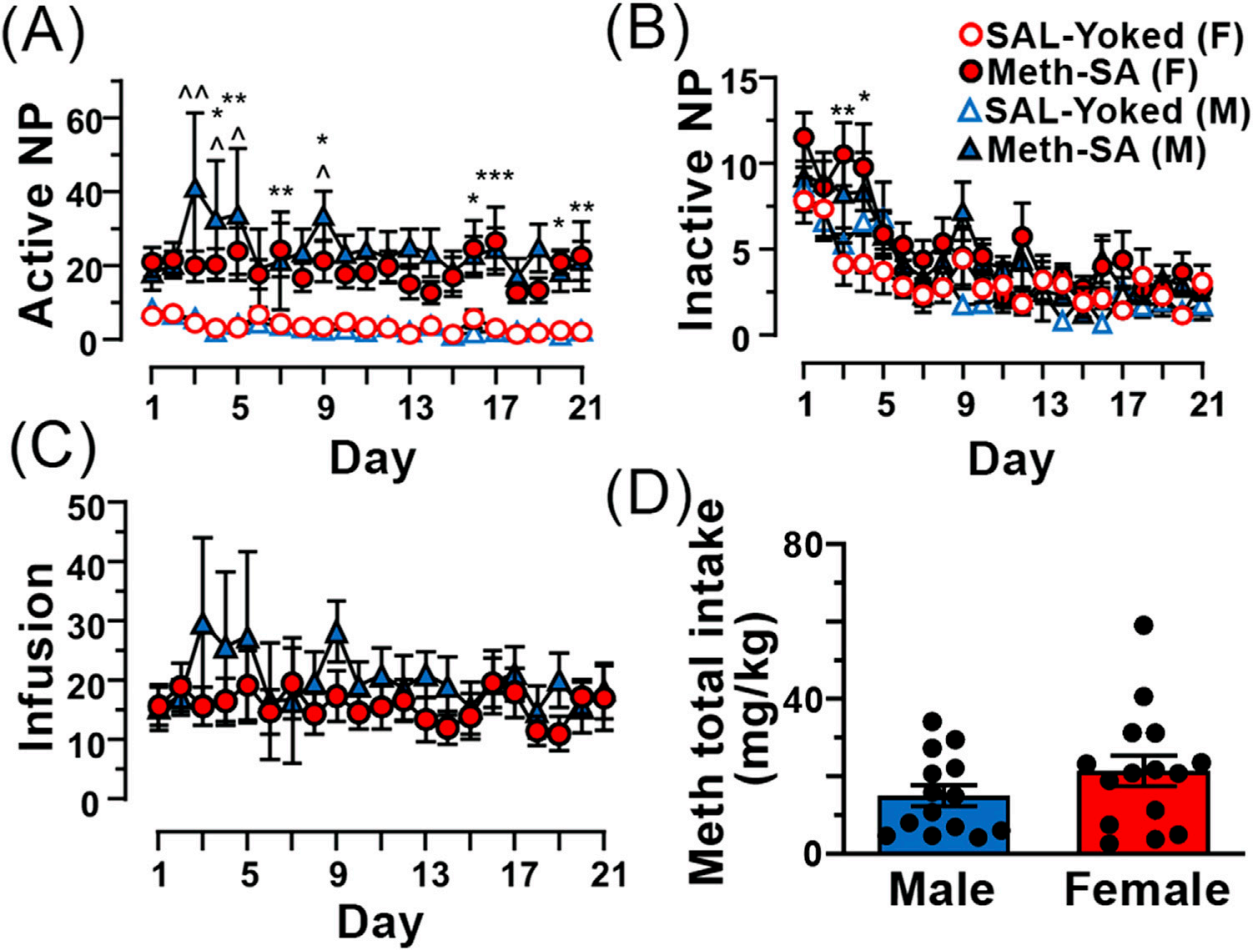
Male and female Meth-SA rats exhibit similar drug-taking behavior. **(A)** The graph shows the average number of daily *active* nose-pokes (NP) across the 21 daily sessions from male (blue filled triangles) and female (red filled circles) Meth-SA and SAL-Yoked (M: blue open triangles, F: red open circles) rats. Statistical analysis: [SAL-Yoked (F) vs. Meth-SA (F): Meth effect: F_(1,27)_ = 29.529, *p <* 0.001; SAL-Yoked (M) vs. Meth-SA (M): Meth effect: F_(1,25)_ = 14.379, *p =* 0.008; SAL-Yoked (F) vs. SAL-Yoked (M): Time effect: F_(20,500)_ = 5.031, *p* < 0.001; Meth-SA (F) vs. Meth-SA (M): *p* > 0.05]. **(B)** The graph shows the average number of daily *inactive* nose-pokes (NP) over the same 21 daily sessions. [SAL-Yoked (F) vs. Meth-SA (F): Meth effect: F_(1,27)_ = 5.945, *p* = 0.022; Time effect: F_(20,540)_ = 6.238, *p* < 0.001; SAL-Yoked (M) vs. Meth-SA (M): Time effect: F_(20,500)_ = 7.598, *p* < 0.001; SAL-Yoked (F) vs. SAL-Yoked (M): Time effect: F_(20,360)_ = 5.276, *p* < 0.001; Meth-SA (F) vs. Meth-SA (M): Time effect: F_(20,540)_ = 8.674, *p* < 0.001]. **(C)** The graph shows the average number of daily Meth infusions across the 21 once-daily session from Meth-SA female (red filled circles) and Meth-SA male (blue filled triangles) rats. **(D)** The graph presents the total Meth intake (mg/kg) over the 21-day Meth-SA sessions for male (blue bar) and female (red bar) Meth-SA rats. Data are represented as MEAN ± SEM. Statistical significance was determined using Two-way rmANOVA to compare Meth-SA behavior **(A–C)** followed by Sidak’s *post hoc* test. Student’s t-test was used to analyze Meth total intake **(D)**. p < 0.05 was considered as statistically significant.

To assess drug-taking behavior in male and female rats, animals were placed in operant chambers (2 h/day) and were monitored for Meth-SA during the 21-day period. Although both female and male rats favored the active nose-poke (NP) in Meth-SA rats compared to SAL-Yoked ones (Figure 2A), no significant sex differences were observed in drug-taking behavior or total Meth intake during the 21-day self-administration period (Figure 2). Both male and female rats showed similar patterns of active nose pokes (Sex Effect: F_(1,27)_ = 0.876, *p* = 0.357) and daily infusions (Sex effect: F_(1,27)_ = 0.654, *p* = 0.426) throughout the protocol (Figures 2A, C). The total Meth intake over the 21-day period did not differ significantly between males (27.8 ± 2.3 mg/kg) and females (29.1 ± 2.1 mg/kg; t_27_ = 0.421, *p* = 0.677) (Figure 2D). This finding contrasts with some previous studies that have reported greater drug intake in females (Roth and Carroll, 2004; Cox et al., 2013; Hartwell et al., 2016), highlighting the complexity of sex differences in drug self-administration.

### Drug-seeking behavior persisted during long-term withdrawal from Meth-SA

**FIGURE 3:**
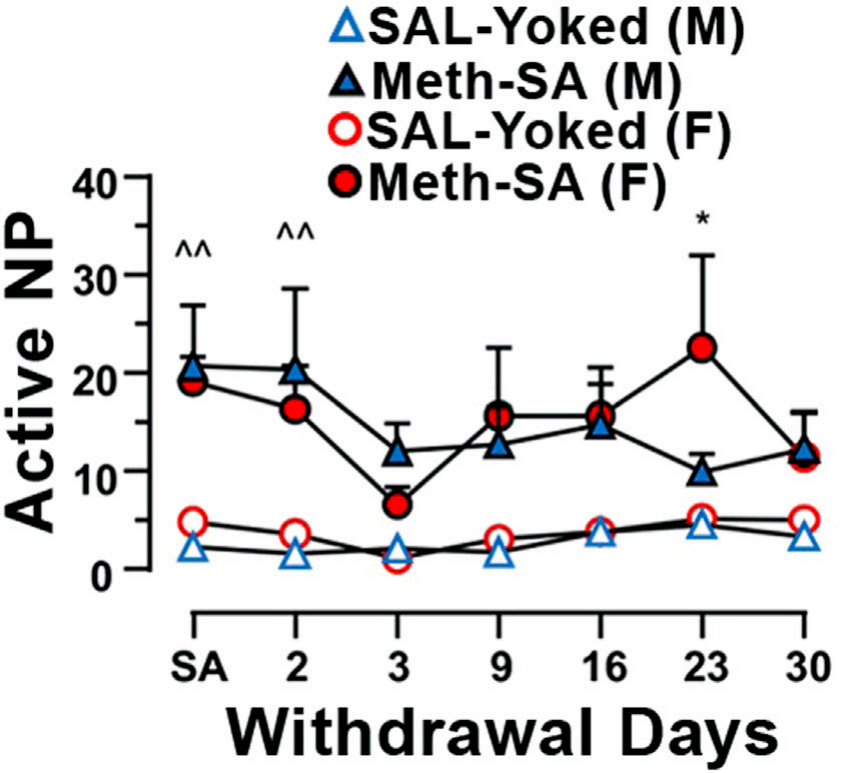
Male and female Meth-SA rats exhibit persistent drug-seeking behavior during a long-term withdrawal. The graph shows the average number of daily active nose-pokes (NP) during the 30-day Meth-SA withdrawal period for male (blue filled triangles) and female (red filled circles), Meth-SA and SAL-Yoked (M: blue open triangles, F: red open circles) rats [SAL-Yoked (M) vs. Meth-SA (M): n = 8 vs. 9. Meth effect: F_(1,15)_ = 9.218, *p* = 0.008; Time effect: F_(6,90)_ = 0.435, *p* = 0.435; Time × Meth: F_(6,90)_ = 1.758, *p* = 0.117; SAL-Yoked (F) vs. Meth-SA (F): n = 8 vs. 10. Meth effect: F_(1,16)_ = 5.539, *p* = 0.032; Time effect: F_(6,96)_ = 2.252, *p* = 0.045; Time × Meth: F_(6,96)_ = 1.020, *p* = 0.417]. **p < 0.01 between Meth-SA (F) and SAL-Yoked (F). ^*p* < 0.05 between SAL-Yoked (M) and SAL-Yoked (M). Data are represented as MEAN ± SEM. Statistical significance was determined using Two-way rmANOVA followed by Sidak’s *post hoc* test. *p* < 0.05 was considered as statistically significant.

Drug-seeking behavior during long-term withdrawal from Meth is a central feature of addiction and remains a significant challenge even after prolonged abstinence. To assess drug-seeking behavior in our Meth-SA rats, we reintroduced them to the operant chambers, but without administering Meth. We measured the frequency of nose-pokes (NPs) as a response to environmental cues previously associated with the drug, which served as an indicator of drug-seeking tendencies. Both male and female Meth-SA rats demonstrated persistent drug-seeking behavior throughout the 30-day withdrawal period (Figure 3). Male Meth-SA rats (blue filled triangles) showed significantly higher active NPs compared to SAL-Yoked controls (Figure 3, blue open triangles) (Meth effect: F_(1,15)_ = 9.218, *p* = 0.008). Similarly, female Meth-SA rats (red filled circles) also exhibited increased drug-seeking behavior (Figure 3) (Meth effect: F_(1,16)_ = 5.539, *p* = 0.032). There was no significant interaction between sex and Meth exposure on drug-seeking behavior (Sex × Meth interaction: F_(1,31)_ = 0.237, *p* = 0.630), suggesting similar persistence of drug-seeking in both sexes.

### Neuronal excitability was significantly reduced in male rats, but not female rats, following short-term withdrawal from Meth-SA

**FIGURE 4:**
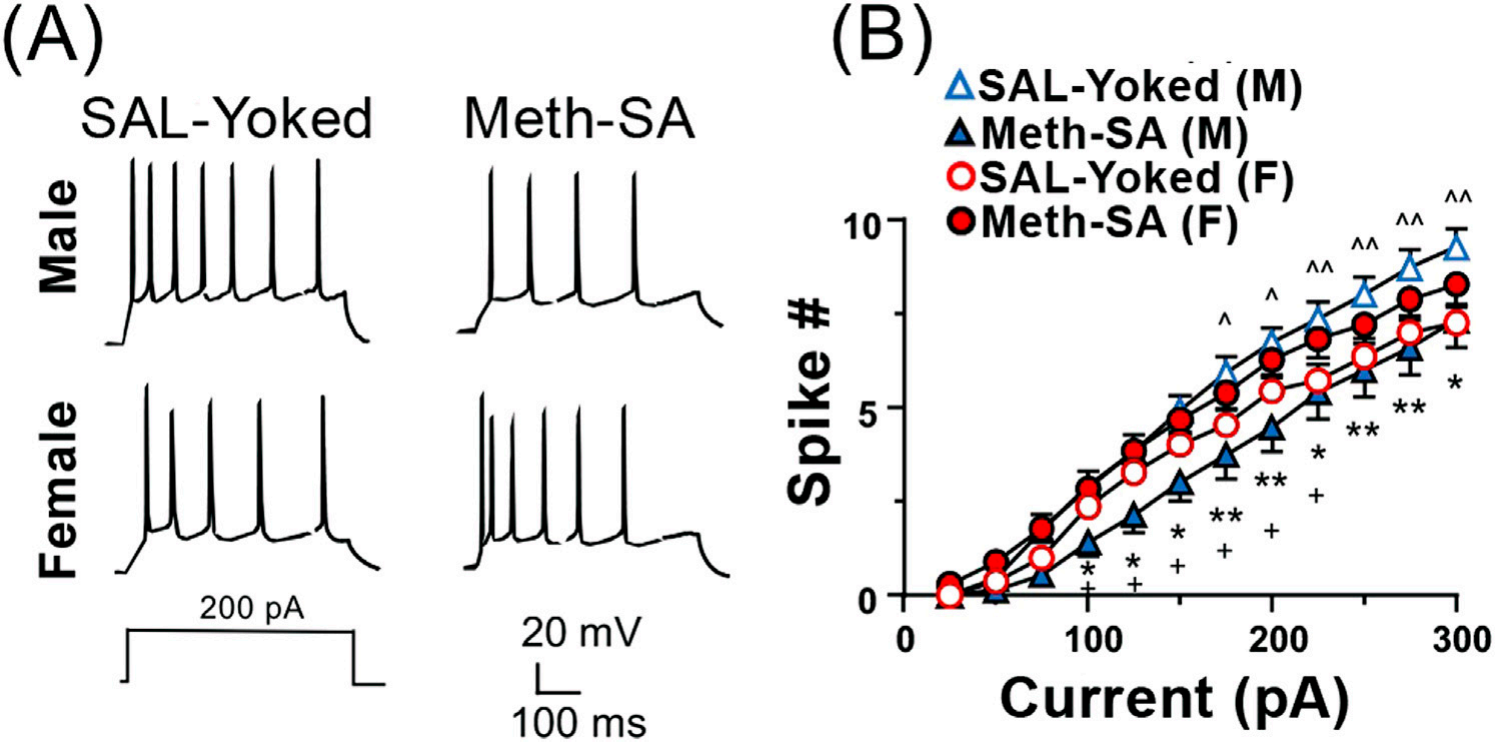
Chronic Meth-SA significantly decreases firing of mPFC pyramidal neurons in male but not female rats following a short-term withdrawal. **(A)** Representative traces of action potential (AP) trains recorded from current clamped mPFC pyramidal neurons, evoked by 500 ms of 200 pA depolarizing current injections in SAL-Yoked vs. Meth-SA male rats (top traces) and SAL-Yoked vs. Meth-SA female rats (bottom traces). Scale bar: 20 mV, 100 msec. **(B)** The graph displays the average number of APs (Spike #) generated during depolarizing current injections (0–300 pA) in SAL-Yoked females (red open circles, n = 11 cells from 4 rats), Meth-SA females (red filled circles, n = 18 cells from 5 rats), SAL-Yoked males (blue open triangles, n = 11 cells from 5 rats) and Meth-SA males (blue filled triangles, n = 15 cells from 5 rats) [SAL-Yoked (F) vs. Meth-SA (F): Current effect: F_(11,297)_ = 255.0, *p* < 0.001; SAL-Yoked (M) vs. Meth-SA (M): Meth effect: F_(1,24)_ = 6.793, *p* = 0.012; Current effect: F_(11,264)_ = 182.9, *p* < 0.001; Current × Meth: F_(11,264)_ = 2.950, *p* = 0.001; SAL-Yoked (F) vs. SAL-Yoked (M): Sex effect: F_(1,10)_ = 7.803, *p* = 0.019; Current effect: F_(11,110)_ = 278.5, *p* < 0.001; Current × Sex F_(11,110)_ = 6.260, *p* < 0.001; Meth-SA (F) vs. Meth-SA (M): Sex effect: F_(1,31)_ = 4.747, *p* = 0.037; Current effect: F_(11,341)_ = 190.9, *p* < 0.001]. *,**p<0.05 or 0.01 between Meth−SA (M) and SAL−Yoked (M). ^∧^,^∧^^∧^,^∧^^∧^^∧^ *p* < 0.05, 0.01 or 0.001 compared between SAL-Yoked (F) and SAL-Yoked (M). +*p* < 0.05 compared between Meth-Yoked (F) and Meth-SA (M). Data are represented as MEAN ± SEM. Statistical significance was determined using Two-way rmANOVA followed by Sidak’s *post hoc* test. *p* < 0.05 was considered as statistically significant.

Previous studies have demonstrated that Meth-SA causes changes in neuronal excitability in the nucleus accumbens (NAc) (Graves et al., 2015) and mPFC (Chen et al., 2012) of male rats. To evaluate potential sex differences in neuronal excitability, we made whole-cell patch clamp recordings from layer V- VI pyramidal neurons in the mPFC of acute cortex slices from male and female Meth-SA rats after short-term withdrawal (2–5 days). We evoked action potential (AP) firing of mPFC pyramidal neurons through 500 msec depolarizing current injections (0–300 pA) (Figure 4A). Consistent with our previous findings (Chen et al., 2012), we found that male Meth-SA rats showed a significant decrease in AP firing rate compared to male SAL-Yoked rats (Figure 4A top traces; Figure 4B) (Meth effect: F_(1,24)_ = 6.793, *p* = 0.012). This decrease in firing was quantified at every 25 pA step of depolarizing current, showing a significantly reduced firing rate (spike #) for all current injections (Figure 4B). Comparisons between male and female SAL-Yoked rats revealed no significant differences in firing rates at any current level, indicating baseline similarities in excitability across sexes. However, unlike males, female Meth-SA rats showed no significant change in firing rate compared to SAL-Yoked female rats (Figure 4A, bottom traces; Figure 4B) (Sex Effect: F_(1,27)_ = 0.237, *p* = 0.630) indicating that sex-specific mechanisms may underlie Meth-induced alterations in neuronal excitability. Additionally, there was no significant difference in spike # for all current injections between male and female SAL-Yoked rats.

### Short-term withdrawal from Chronic Meth-SA induced reduction in calcium influx via VGCCs in mPFC pyramidal neurons in male rats, but an increase in female rats

**FIGURE 5:**
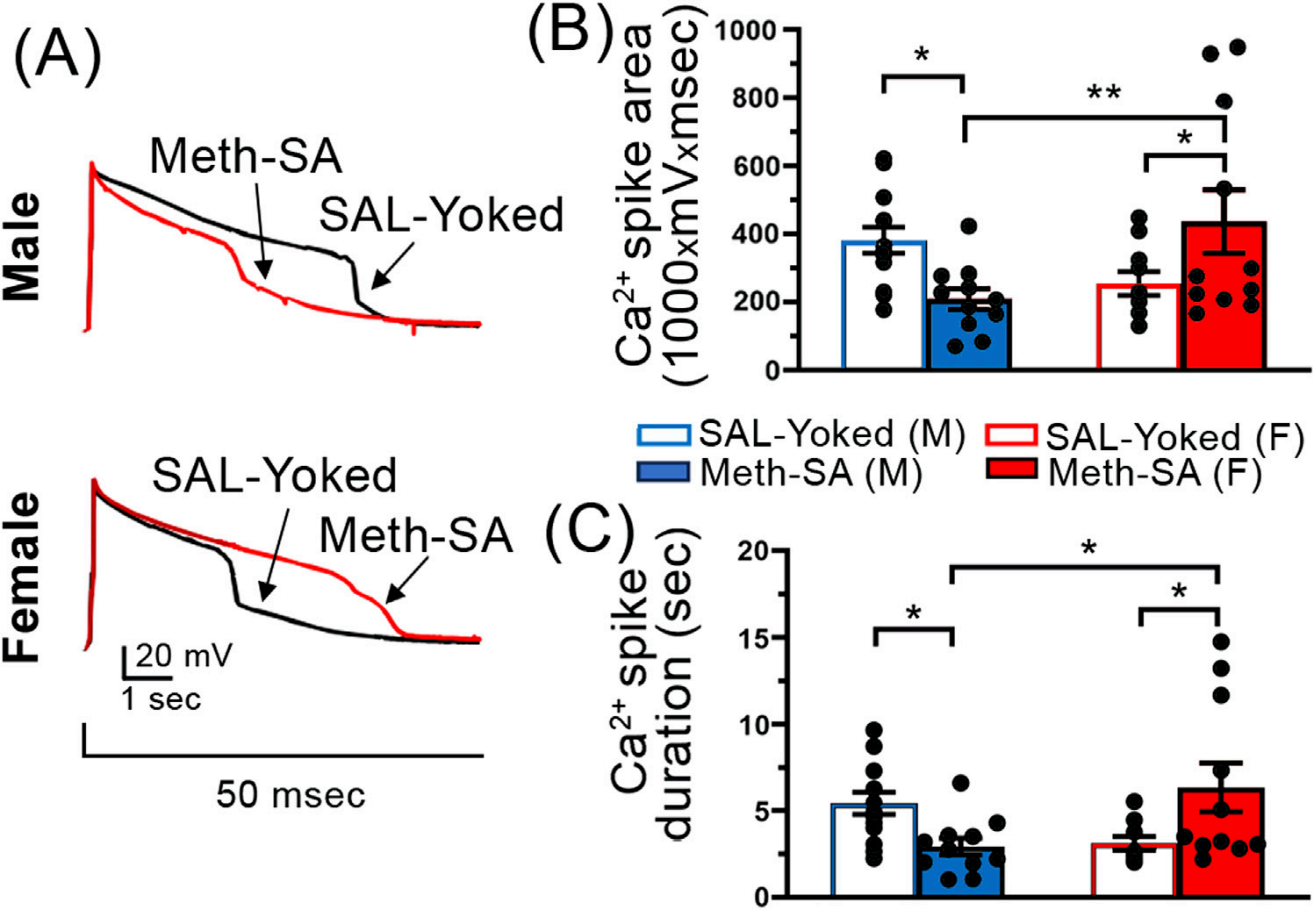
Sex-specific alterations in Ca^2+^ spikes in mPFC pyramidal neurons following a short-term withdrawal from Meth-SA. Chronic Meth-SA followed by a short-term withdrawal induced a significant reduction in calcium influx via VGCCs among mPFC pyramidal neurons in male rats; meanwhile it caused an increase in female rats. **(A)** Representative traces of Ca^2+^ spikes recorded from mPFC pyramidal neurons in response to 50 msec depolarization in SAL-Yoked (black traces) vs. Meth-SA (red traces) in male (top traces) and female (bottom traces) rats. Scale Bar: 20 mV, 1 s. **(B)** The graph shows the average Ca^2+^ spike area (measured as area under the curve, 1,000 × mV × msec) in SAL-Yoked males (blue open bars, n = 13 cells from 7 rats) and Meth-SA males (blue filled bars, n = 11 cells from 7 rats), SAL-Yoked females (red open bars, n = 10 cells from 5 rats) and Meth-SA females (red filled bars, n = 11 cells from 5 rats) [SAL-Yoked (M), Meth-SA (M), SAL-Yoked (F) vs. Meth-SA (F): n = 13, 11, 10 vs. 11. Meth effect: F_(1,41)_ = 0.007, *p* = 0.932; Sex effect: F_(1,41)_ = 0.825, *p* = 0.369; Sex × Meth: F_(1,41)_ = 10.30, *p* = 0.003]. **(C)** The graph shows the average Ca^2+^ spike duration (sec) in SAL-Yoked (white bars) and Meth-SA (red bars) male (bars on the left) and female (bars on the right) rats [SAL-Yoked (M), Meth-SA (M), SAL-Yoked (F) vs. Meth-SA (F): n = 13, 11, 9 vs. 11. Meth effect: F_(1,40)_ = 0.183, *p* = 0.671; Sex effect: F_(1,40)_ = 0.420, *p* = 0.521; Sex × Meth: F_(1,40)_ = 11.11, *p* = 0.002]. Data are represented as MEAN ± SEM. Statistical significance was determined using Two-way ANOVA followed by Sidak’s *post hoc* test. *p* < 0.05 was considered as statistically significant.

Our data show that male and female rats exhibited divergent changes in mPFC pyramidal neuron excitability following short-term (2–5 days) withdrawal from Meth-SA (Figure 4). Voltage-gated calcium channels (VGCCs) play a crucial role in the regulation of neuronal excitability by modulating the flow of Ca^2^⁺ into the cell in response to membrane depolarization. Several animal studies indicate VGCC dysregulation as a result of Meth exposure (Shibasaki et al., 2010; Andres et al., 2015). To assess VGCC function, we performed whole-cell patch clamp recordings of mPFC pyramidal neurons in the presence of appropriate inhibitors/antagonists that block other membrane ion channels (see Methods Section) and elicited Ca^2+^ plateau potentials by a minimal depolarizing current injection. We found that VGCC function, as assessed by the area and duration of evoked calcium spikes, also showed sex-specific alterations during short-term withdrawal (Figure 5). Male Meth-SA rats exhibited a significant decrease in calcium spike area (Meth effect: t_22_ = 2.743, *p* = 0.012) and duration (Meth effect: t_22_ = 2.956, *p* = 0.007) compared to SAL-Yoked controls (Figure 5A, top trace, 5B, 5C). Conversely, female Meth-SA rats showed a significant increase in both calcium spike area (Meth effect: t_19_ = 2.518, *p* = 0.021) and duration (Meth effect: t_18_ = 2.392, *p* = 0.028) compared to controls (Figure 5A (bottom trace, 5B, 5C). A two-way ANOVA revealed a significant interaction between sex and Meth exposure for both calcium spike area (Sex × Withdrawal interaction: F_(1,41)_ = 10.30, *p* = 0.003) and duration (Sex × Withdrawal interaction: F_(1,40)_ = 11.11, *p* = 0.002). These opposing effects between Meth-SA males and females are consistent with previous studies showing sex differences in neuroadaptations during early withdrawal from other drugs of abuse (Becker et al., 2012).

### Neuronal hyperactivity occurred after long-term withdrawal from Meth-SA regardless of sex

**FIGURE 6:**
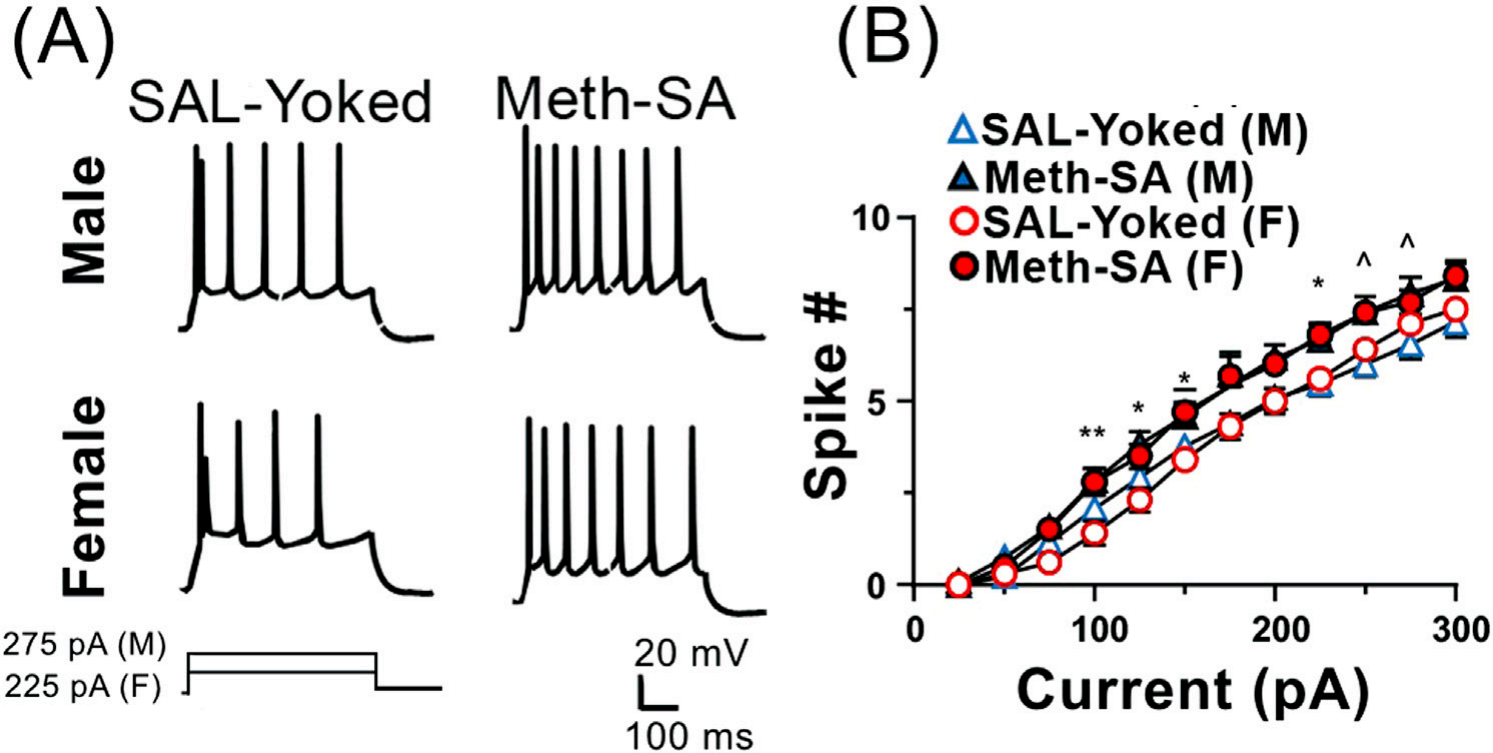
Firing of mPFC pyramidal neurons is significantly increased in Meth-SA rats following a long-term withdrawal, regardless of sex. **(A)** Representative traces of AP trains recorded from current clamped mPFC pyramidal neurons, evoked by 500 ms of 275 pA (male) and 225 pA (female) depolarizing current injections in SAL-Yoked vs. Meth-SA female rats (F, top traces) and SAL-Yoked vs. Meth-SA male rats (M, bottom traces). Scale bar: 20 mV, 100 msec. **(B)** The graph displays the average number of APs (Spike #) generated during depolarizing current injections (0–300 pA) in SAL-Yoked females (red open circles, n = 10 cells from 4 rats), Meth-SA females (red filled circles, n = 10 cells from 6 rats), SAL-Yoked males (blue open triangles, n = 13 cells from 4 rats) and Meth-SA males (blue filled triangles, n = 12 cells from 4 rats) [SAL-Yoked (M) vs. Meth-SA (M): n = 13 vs. 12. Meth effect: F_(1,23)_ = 5.637, *p* = 0.026; Current effect: F_(11,253)_ = 354.7, *p* < 0.001; Current × Meth: F_(11,253)_ = 2.057, *p* = 0.024; SAL-Yoked (F) vs. Meth-SA (F): n = 10 vs. 10. Meth effect: F_(1,18)_ = 7.817, *p* = 0.012; Current effect: F_(11,198)_ = 454.6, *p* < 0.001; Current × Meth: F_(11,198)_ = 2.705, *p* = 0.003]. *^,^**p < 0.05 or 0.01 between Meth-SA (F) and SAL-Yoked (F). ^*p* < 0.05 between SAL-Yoked (M) and SAL-Yoked (M). Data are represented as MEAN ± SEM. Statistical significance was determined using Two-way rmANOVA followed by Sidak’s *post hoc* test. *p* < 0.05 was considered as statistically significant.

Meth, like other drugs of abuse, can induce long-lasting neuronal changes that persist even during long-term withdrawal. To investigate potential sex differences in these enduring changes, we repeated patch-clamp experiments and measured neuronal firing in response to depolarizing currents in both male and female Meth-SA rats after approximately 30 days of withdrawal. Unlike with short-term withdrawal, our results showed a significant increase in the firing rates of mPFC pyramidal neurons in both male (top traces) and female (bottom traces) Meth-SA rats compared to saline controls (Figure 6A). Quantification at every 25 pA step of depolarizing current demonstrated a significantly increased firing rate (spike #) for multiple current injections in both male and female Meth-SA rats (Figure 6B). Post-*hoc* statistical analysis indicated no significant interaction between sex and meth exposure on neuronal firing (Sex × Meth interaction: F_(1,41)_ = 0.561, *p* = 0.459), suggesting that long-term Meth withdrawal leads to increased neuronal excitability in the mPFC, independent of sex. This enhanced excitability may underlie the persistent behavioral and cognitive challenges observed following prolonged Meth use.

### Calcium influx was abnormally increased in mPFC neurons in both male and female rats after long-term withdrawal from Meth-SA

**FIGURE 7:**
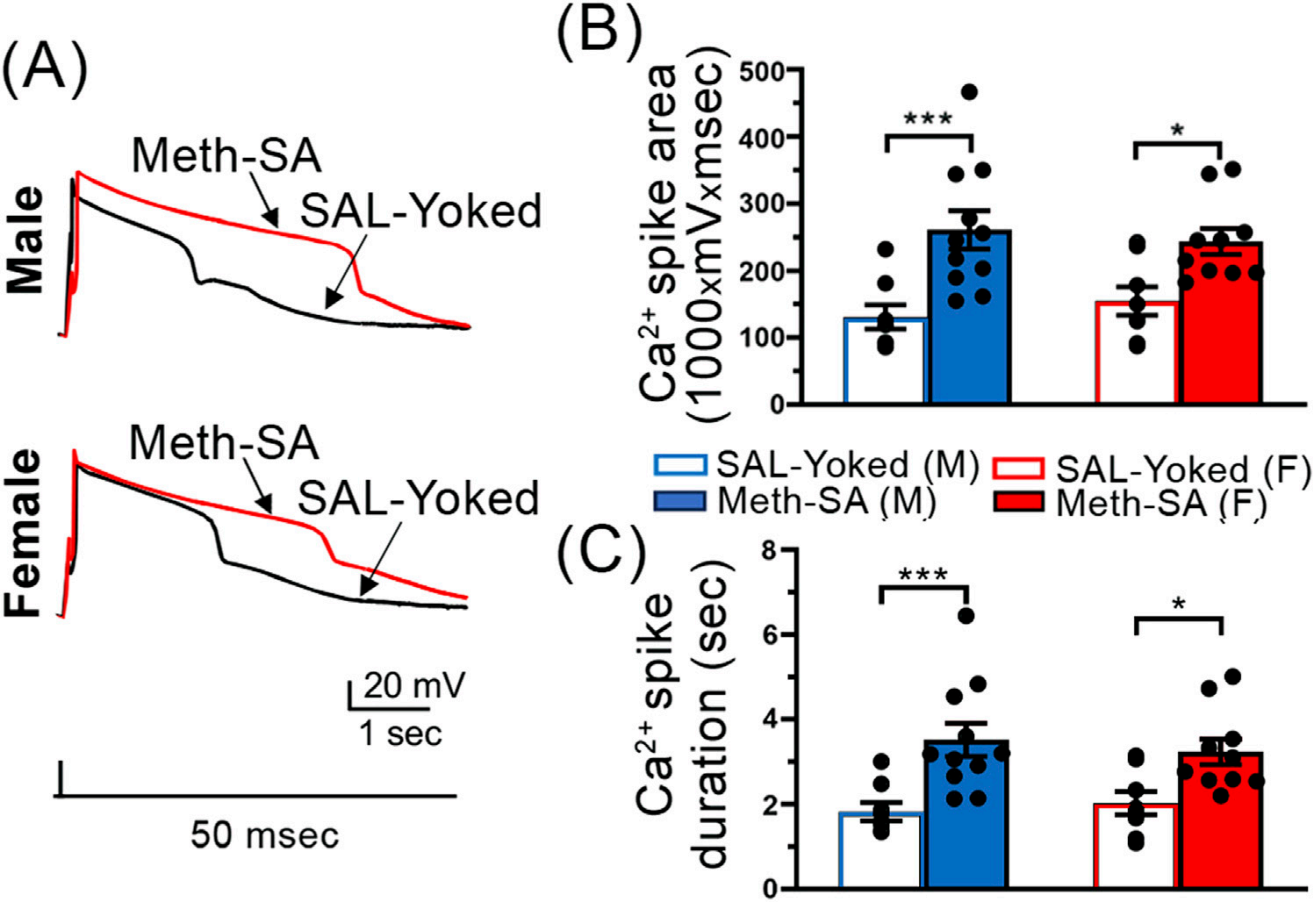
Ca^2^⁺ spikes in mPFC pyramidal neurons are significantly prolonged in Meth-SA rats following a long-term withdrawal, regardless of sex. **(A)** Representative traces of Ca^2+^ spikes recorded from mPFC pyramidal neurons in response to 50 msec depolarization in SAL-Yoked (black traces) vs. Meth-SA (red traces) in male (top trace) and female (bottom trace) rats. Scale Bar: 20 mV, 1 s. **(B)** The graph shows the average Ca^2+^ spike area (measured as area under the curve, 1,000 × mV × msec) in SAL-Yoked males (blue open bars, n = 8 cells from 6 rats) and Meth-SA males (blue filled bars, n = 10 cells from 5 rats), SAL-Yoked females (red open bars, n = 8 cells from 5 rats) and Meth-SA females (red filled bars, n = 11 cells from 7 rats) [SAL-Yoked (M), Meth-SA (M), SAL-Yoked (F) vs. Meth-SA (F): n = 8, 11, 8 vs. 10. Meth effect: F_(1,33)_ = 19.89, *p* < 0.001; Sex effect: F_(1,33)_ = 0.013, *p* = 0.910; Sex × Meth: F_(1,33)_ = 0.561, *p* = 0.459]. **(C)** The graph shows the average Ca^2+^ spike duration (sec) in SAL-Yoked (white bars) and Meth-SA (red bars) for male (left bars) and female (right bars) rats. [SAL-Yoked (M), Meth-SA (M), SAL-Yoked (F) vs. Meth-SA (F): n = 8, 11, 8 vs. 10. Meth effect: F_(1,33)_ = 21.69, *p* < 0.001; Sex effect: F_(1,33)_ = 0.019, *p* = 0.890; Sex × Meth: F_(1,33)_ = 0.762, *p* = 0.389]. Data are represented as MEAN ± SEM. Statistical significance was determined using Two-way ANOVA followed by Sidak’s *post hoc* test. *p* < 0.05 was considered as statistically significant.

To investigate if the observed increase in neuronal firing during long-term withdrawal corresponds with changes in VGCC function, we assessed Ca^2+^ influx in mPFC neurons in male and female Meth-SA rats following 30-day withdrawal. Unlike during short-term withdrawal our data suggests that VGCC function was similarly altered in both sexes following long-term withdrawal from Meth (red traces, Figures 7A–C). Both male (top trace) and female (bottom trace) Meth-SA rats showed significantly increased calcium spike area (Figure 7B) (Meth effect: F_(1,33)_ = 19.89, *p* < 0.001) and duration (Figure 7C) (Meth Effect: F_(1,33)_ = 21.69, *p* < 0.001) compared to SAL-Yoked controls. Post-*hoc* analysis revealed no significant differences between males and females in the magnitude of these increases. These results demonstrate that Meth-SA induces sex-specific neuroadaptations in mPFC pyramidal neurons during short-term withdrawal, which converge to similar alterations following long-term withdrawal. These neuronal changes are accompanied by persistent drug-seeking behavior in both sexes (Figure 3).

### Environment affected voltage-sensitive Ca^2+^ influx in mPFC pyramidal neurons

**FIGURE 8:**
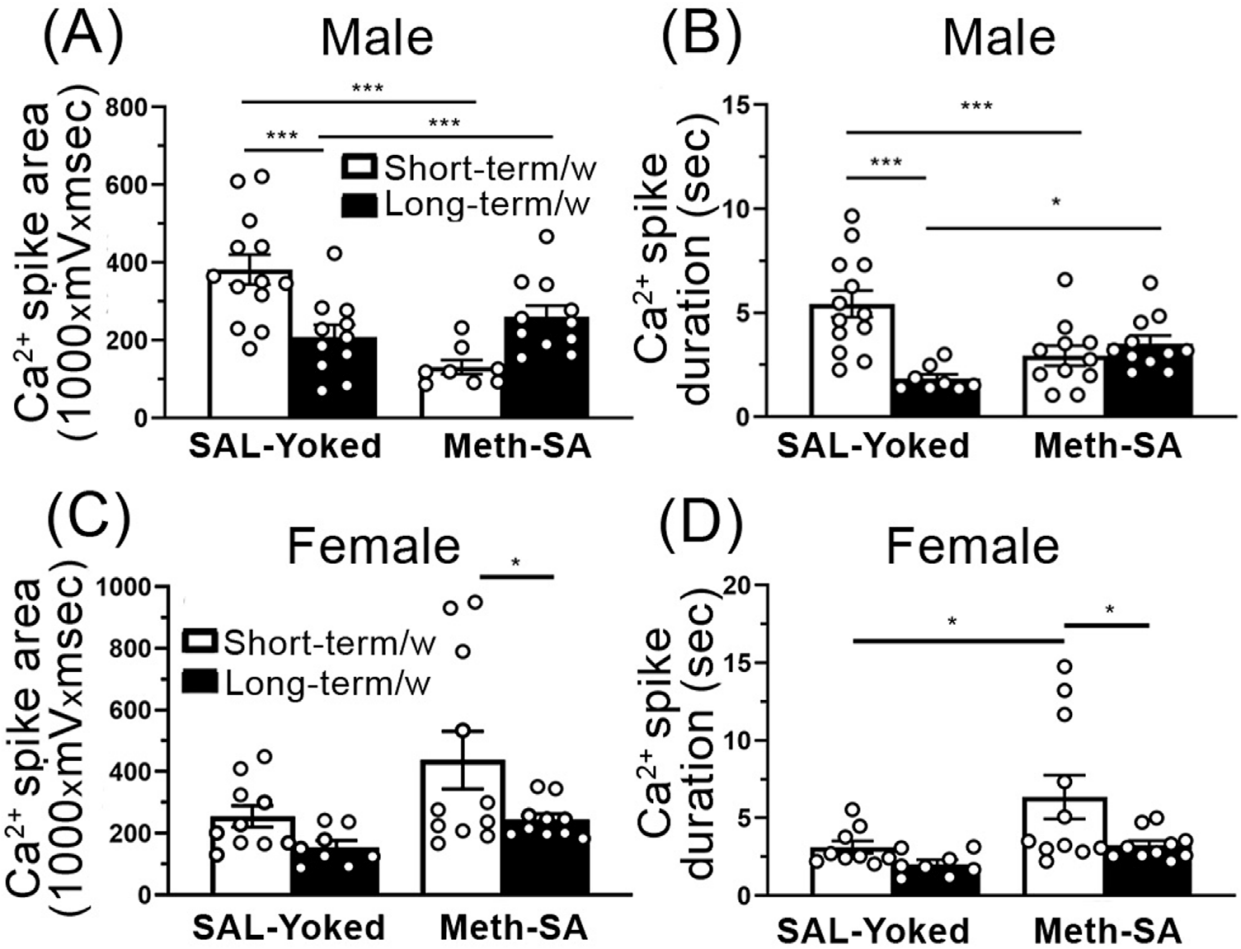
Ca^2^⁺ influx via VGCCs is reduced in mPFC pyramidal neurons in SAL-Yoked male, but not SAL-Yoked female rats, after a long-term withdrawal. **(A, B)** The graphs show average Ca^2+^ spike area **(A)** (measured as the area under the curve, 1,000 × mV × msec) and Ca^2+^ spike duration **(B)** in SAL-Yoked males (left bars) and Meth-SA males (right bars) after a short-term withdrawal (white bars) and long-term withdrawal (black bars). In SAL-yoked (M) male rats, both Ca^2^⁺ spike area and duration were significantly decreased following long-term withdrawal compared to short-term withdrawal [SAL-Yoked (M) Short-term, Long-term, Meth-SA(M) Short-term, Long-term, Area: WD effect: F_(1,39)_ = 9.218, *p* = 0.004; Meth effect: F_(1,39)_ = 0.426, *p* = 0.518; interaction: F_(1,39)_ = 21.22, *p* < 0.001; Duration: F_(1,39)_ = 8.560, *p* = 0.006; Meth effect: F_(1,39)_ = 0.604, *p* = 0.442; interaction: F_(1,39)_ = 16.39, *p* < 0.001]. **(C, D)** The graphs show average Ca^2+^ spike area **(C)** and Ca^2+^ spike duration **(D)** in SAL-Yoked females (left bars) and Meth-SA females (right bars) after a short-term withdrawal (white bars) and long-term withdrawal (black bars). No significant differences were detected in Ca^2^⁺ spikes measured in Meth-SA male or female, nor in SAL-yoked female rats across withdrawal periods [SAL-Yoked (F) Short-term, Long-term, Meth-SA (F) Short-term, Long-term, Area: WD effect: F_(1,35)_ = 6.443, *p* = 0.016; Meth effect: F_(1,35)_ = 5.501, *p* = 0.025; interaction: F_(1,35)_ = 0.652, *p* = 0.425; Duration: WD effect: F_(1,34)_ = 5.838, *p* = 0.021; Meth effect: F_(1,34)_ = 6.472, *p* = 0.016; interaction: F_(1,34)_ = 1.349, *p* = 0.254]. All data are represented as MEAN ± SEM. Statistical significance was determined using Two-way ANOVA followed by Sidak’s *post hoc* test. *p* < 0.05 was considered as statistically significant.

We assessed the influences of environment, as well as the effect of Meth-SA on Ca^2^⁺ influx via VGCCs in mPFC pyramidal neurons in male and female rats following short-term and long-term withdrawal periods. We found that in male rats, SAL-Yoked animals showed a significant reduction in Ca^2^⁺ spike area (Figure 8A) and duration (Figure 8B) in long-term withdrawal compared to short-term withdrawal. However, in Meth-SA males, no significant differences in Ca^2^⁺ spike area (Figure 8A) or duration (Figure 8B) were observed between the short-term and long-term withdrawal conditions, indicating a possible stabilizing effect of Meth-SA on VGCC-mediated calcium influx over time. In female rats, SAL-Yoked groups did not display significant changes in Ca^2^⁺ spike area (Figure 8C) or duration (Figure 8D) between short-term and long-term withdrawal conditions. However, we found that Meth-SA female treated rats displayed a significant decrease in Ca^2^⁺ spike area (Figure 8C) and duration (Figure 8D) after long-term withdrawal. This demonstrates that restrained movement in a closed environment could affect the neuronal activity via reduction in Ca^2+^ influx in both SAL-Yoked and Meth-SA treated animals.

## Discussion

The present study assessed the sex differences in Meth use by examining functional changes in the mPFC neurons of rats following both short-term and long-term withdrawal from Meth-SA. Our findings provide valuable insights into the sex-specific mechanisms underlying Meth-induced changes in neuronal excitability and drug-seeking behavior, highlighting the critical role of sex as a biological variable in addiction and relapse.

### Both male and female rats self-administered meth

Our results indicate that both male and female rats exhibited similar patterns of active nose pokes (drug-taking behaviors) and daily Meth infusions during the 21-day self-administration period, with no statistically significant sex differences in drug-taking behavior or total Meth intake (Figures 2A–C). However, a trend toward increased Meth intake was observed in female rats (Figure 2D). This finding seems to be in agreement with previous studies that reported higher Meth consumption in female rats compared to males (Roth and Carroll, 2004; Cox et al., 2013; Hartwell et al., 2016) while the discrepancy between our findings and prior research are likely due to differences in experimental design. Importantly, our results are consistent with other studies using comparable Meth-SA protocols, which also found no significant sex differences in Meth intake (Johansen and McFadden, 2017; Venniro et al., 2017; Pena-Bravo et al., 2019). Thus, sex differences in Meth intake may be more nuanced and dependent on experimental conditions.

During the long-term withdrawal phase (30 days), both male and female Meth-SA rats displayed persistent drug-seeking behavior, as evidenced by the increased frequency of active nose pokes in response to environmental cues previously associated with Meth (Figure 3). This persistence of drug-seeking behavior suggests that the reinforcing effects of Meth and the neuroadaptations that occur during Meth use can drive similar relapse-like behavior in both sexes. This observation supports the notion that Meth addiction induces long-term neuroplastic changes that sustain drug-seeking behavior, regardless of sex. These findings highlight the complexity of sex differences in Meth-SA and suggest that, while both sexes may exhibit similar drug-seeking behavior after long-term withdrawal, subtle sex-specific factors may still influence Meth intake and associated behaviors.

### Distinct neuronal dysfunction in male and female rats following short-term withdrawal from Meth-SA

One of the key findings in the present study is the identification of sex-specific changes in neuronal function following a short-term withdrawal from Meth. Our data demonstrate a decrease in the firing rate of mPFC pyramidal neurons in male rats, but not in females (Figure 4). Although previous studies have shown a reduction in mPFC pyramidal neuron excitability after short-term Meth withdrawal in both male mice and rats (Chen et al., 2012; Leyrer-Jackson et al., 2021), we are, to the best of our knowledge, the first to report that this reduction is sex-specific, occurring only in males. One putative mechanism underlying the sex-divergent firing frequency between male and female rats in mPFC pyramidal neurons could be alterations in VGCCs. To that end, our findings reveal sex-specific differences in VGCC function (Figure 5): male Meth-SA rats exhibited a significant decrease in calcium spike area (Figure 5B) and duration (Figure 5C), whereas female Meth-SA rats showed significant increases in these parameters (Figure 5A). The disconnect between Ca^2^⁺ influx and firing rate suggests that females may employ additional regulatory mechanisms to maintain excitability, even under conditions of altered calcium dynamics mediated by VGCCs. This finding highlights the potential for compensatory or sex-specific responses in females that help buffer against Meth-SA induced neurophysiological changes.

VGCCs play a critical role in the regulation of neuronal excitability by allowing the flow of Ca^2^⁺ into neurons in response to membrane depolarization. This Ca^2^⁺ influx is essential for various neuronal functions, including synaptic transmission, neurotransmitter release, and the modulation of action potential firing. In our study, the observed decrease in Ca^2^⁺ current (both in terms of area and duration) in the mPFC of male Meth-SA rats after a short-term withdrawal could at least in part, explain the reduced firing rate of pyramidal neurons. Reduced Ca^2^⁺ influx could impair the ability of these neurons to generate sufficient depolarization to reach the threshold for firing action potentials, contributing to the overall decrease in excitability observed in male rats during short-term Meth withdrawal. These findings are consistent with prior research showing that Meth exposure can dysregulate calcium signaling, either by reducing calcium channel function or by affecting the balance between excitatory and inhibitory neurotransmission (Andres et al., 2015; González et al., 2016). In our study, the sex-specific response, with males showing decreased calcium currents, suggests that VGCC dysfunction is a key contributor to the Meth-induced neuronal dysfunctions in males following a short-term withdrawal, providing a potential mechanism for the differential neurophysiological responses between sexes. This VGCC-mediated dysregulation could be a critical factor in the persistent cognitive and behavioral deficits observed following Meth use, as Ca^2^⁺ signaling is crucial for maintaining synaptic plasticity and proper cortical function.

In addition to VGCCs, alterations in synaptic transmission may also contribute to the observed sex-specific changes in mPFC excitability. Our data demonstrating the decrease in pyramidal neuron excitability in males aligns with existing literature that points to alterations in GABAergic transmission in the mPFC following Meth use. Specifically, the observed reduction in excitability may be driven by increases in inhibitory postsynaptic current (IPSC) amplitude and frequency in male rats after a 7-day Meth abstinence period (Andres et al., 2015; Armenta-Resendiz et al., 2022; 2024). This suggests a shift in the excitation/inhibition (E/I) balance, leading to reduced neuronal excitability. Interestingly, this effect appears to be sex-specific, as female rats did not exhibit increased IPSC amplitude (but did exhibit increase in IPSC frequency), which could explain the absence of reduced excitability in females and suggests distinct sex-specific neuroadaptations in response to Meth exposure.

Dopamine D1/D5 receptors (DR1/5) on GABAergic neurons in the mPFC are crucial in regulating neuronal excitability and synaptic transmission, especially in the context of Meth exposure and addiction (González et al., 2016; Anastasiades et al., 2019; Armenta-Resendiz et al., 2022; Daiwile et al., 2022). Meth increases dopamine levels in the mPFC, overstimulating DR1/5 receptors on both pyramidal neurons and GABAergic interneurons. This over stimulation disrupts inhibitory signaling, impairing mPFC function, which in turn contributes to compulsive drug-seeking behaviors and cognitive deficits often observed in Meth addiction (Daiwile et al., 2022). Furthermore, Meth-induced DR1/5 receptor overstimulation exacerbates synaptic imbalances by affecting AMPA and NMDA receptor expression, as previously reported (González et al., 2016), leading to disrupted neural circuits involved in addiction. Future studies should delve deeper into these sex-specific mechanisms, exploring how alterations in VGCC function and synaptic transmission interact to regulate mPFC excitability and how these processes differ between males and females following Meth exposure. Understanding these complex pathways could provide valuable insights into developing sex-specific therapeutic strategies.

### Similar neuronal dysfunction in both male and female rats after long-term withdrawal from Meth-SA

Studying the neuronal changes that occur during and after long-term withdrawal from Meth is essential for understanding the persistent effects of drug abuse on the brain. Unlike the patterns observed during short-term withdrawal, we found a significant increase in the firing rates of mPFC pyramidal neurons in both male and female Meth-SA rats after approximately 30 days of withdrawal (Figure 6). This heightened excitability is likely linked to the behavioral and cognitive challenges often observed after prolonged Meth use. The increase in neuronal excitability in both sexes was accompanied by elevated Ca^2^⁺ influx, as shown by a significant rise in Ca^2^⁺ spike area and duration in mPFC pyramidal neurons compared to saline (SAL-Yoked) controls (Figure 7). These findings indicate that Meth-SA induces divergent sex-specific neuroadaptations during short-term withdrawal, which later converge into similar excitability profiles during long-term withdrawal. The increased excitability we observed after long-term Meth withdrawal aligns with findings from studies on other drugs of abuse, such as cocaine, where prolonged withdrawal is also associated with enhanced firing rates in the mPFC (Nasif et al., 2005a; 2005b; Wayman et al., 2015; 2016). The convergence in neuronal changes between sexes during long-term withdrawal suggests that, although different mechanisms may be employed initially, Meth use leads to similar long-term adaptations in the mPFC. In addition, this finding also suggests that long-term withdrawal from chronic Meth-SA might diminish a potential self-protecting mechanism (that was activated during short-term withdrawal) to reduce neuronal hyperactivity in Meth-SA male rats. In contrast, Meth-SA female rats may lack such a mechanism and therefore, could be more vulnerable than males in response to chronic Meth exposure.

**FIGURE 9:**
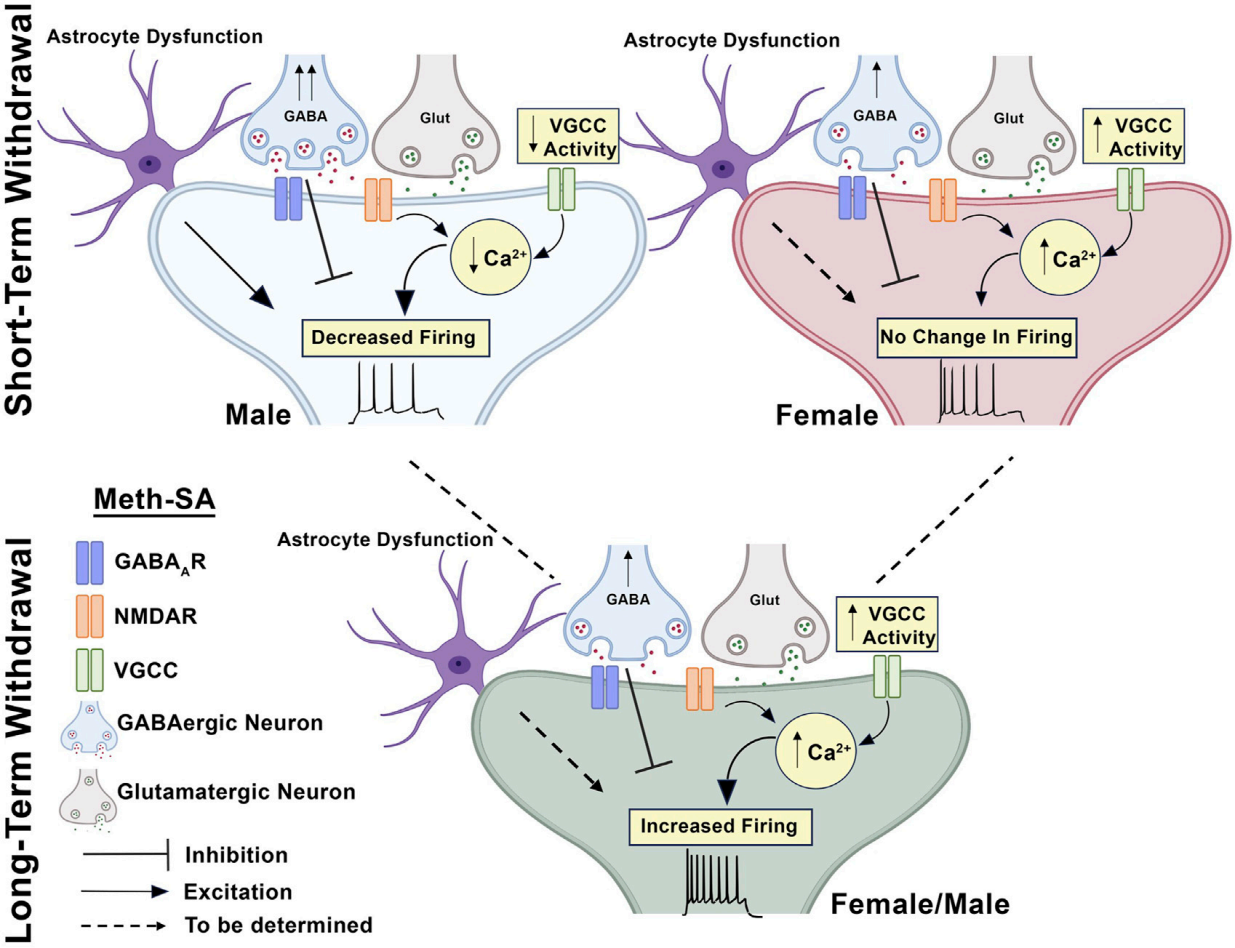
Chronic Meth-SA followed by a short-term or long-term withdrawal distinctively alters the functional activity of mPFC pyramidal neurons in male and female rats. Short-Term Withdrawal (Top Panel): Male Neurons (Left): In male rats, short-term withdrawal from Meth-SA leads to a significant decrease in firing activity of mPFC pyramidal neurons. This reduction in neuronal excitability is linked to a significant decrease in Ca^2^⁺ influx, as observed through reduced VGCC activity. Astrocyte dysfunction, indicated by disrupted astrocytic support of neuronal function, may also contribute to the diminished neuronal activity observed. Prior research also has shown that Meth withdrawal in males results in altered GABAergic synaptic transmission, which further disrupts the excitation-inhibition balance in the mPFC. Female Neurons (Right)*:* In female rats, short-term withdrawal from Meth-SA does not induce significant changes in the firing activity of mPFC pyramidal neurons compared to saline (SAL)-Yoked controls, despite an observed increase in intracellular Ca^2^⁺ levels. Long-Term Withdrawal (Bottom Panel): Both Sexes (Green Neuron): After long-term withdrawal (≥30 days) from Meth-SA, both male and female rats exhibit similar increase in firing activity of mPFC pyramidal neurons compared to SAL-Yoked controls. This abnormal neuronal hyperactivity corresponds with an increase in Ca^2^⁺ influx through VGCCs, indicating that such prolonged Meth withdrawal induces similar neuroadaptive hyperactivity across sexes. Although astrocyte dysfunction has not been tested in this context, it is very likely that its effects also persist, altering the synaptic environment and potentially contributing to the abnormal increase in mPFC neuronal firing activity.

Recent research by Hu et al. (2024) has demonstrated that Meth-associated memory retrieval activates inhibitory neurons in the mPFC, facilitating memory reconsolidation. Concurrently, excitatory neurons are suppressed, which likely aids in the expression of these addictive memories. These findings suggest a complex interplay between excitatory and inhibitory neuronal circuits in the mPFC during the recall of drug-related memories, potentially contributing to the persistence of addiction behaviors during withdrawal (Hu et al., 2024). Another possible factor contributing to these persistent changes is interactive astrocyte-neuron dysfunction. Astrocytes play a critical role in supporting neuronal health, modulating synaptic activity, and maintaining homeostasis in the mPFC. Our previous study has demonstrated that astrocyte function is significantly disturbed following acute exposure to Meth, which could exacerbate dysfunction of cortical pyramidal neurons nearby (Dave et al., 2019). Our recent study also demonstrates an astrocyte dysfunction that occurs concurrently with mPFC neuronal dysfunction following exposure to chronic cocaine (in preparing for publication) (Figure 9). Dysregulation in astrocyte-neuron interactions could further compromise the excitatory/inhibitory balance and impair the mPFC’s resilience to Meth, potentially exacerbating cognitive and behavioral symptoms during withdrawal. Investigating astrocyte dysfunction as it relates to both short- and long-term withdrawal could provide insights into additional therapeutic targets.

### Influences of environment on neuronal Ca^2+^ influx in SAL-Yoked male rats

Our data also demonstrate a divergent pattern of Ca^2+^ influx in SAL-Yoked, but not Meth-SA male rats, following a short-term and long-term withdrawal. Conversely, our data demonstrate a lack of Ca^2+^ change in SAL-Yoked female rats but a decrease in Ca^2+^ response following long-term withdrawal in Meth-SA treated female rats (Figure 8). This decrease of Ca^2^⁺ influx through VGCCs (regardless of sex or drug influence) among glutamatergic cortical pyramidal neurons in the brains of male and female rats suggests that repeated and prolonged limitation of movement in a small and enclosed environment could not only restrain unmotivated behaviors mediated by the motor cortex, but also diminish certain cognitive functions mediated by the mPFC due to disrupted neuronal calcium homeostasis (e.g., a significant decrease in intracellular calcium level).

## Summary

This study examined sex differences in Meth-induced changes in the mPFC of rats, focusing on both short-term and long-term withdrawal effects. Our novel findings reveal distinct male-female differences in neuronal adaptations: following short-term withdrawal, male rats showed reduced firing rates of mPFC neurons and decreased calcium influx through VGCCS, while females exhibited increases in calcium spike area and duration, suggesting divergent calcium homeostatis and neurophysiological responses. In contrast, long-term withdrawal led to converging patterns, with both sexes showing abnormally increased neuronal excitability and calcium influx (Figure 9). This sustained hyperexcitability may underlie persistent behavioral and cognitive challenges associated with Meth addiction and MUD, including heightened drug-seeking behavior, increased risk of relapse, and long-term cognitive deficits. Clinically, our results underscore the importance of sex-specific approaches to MUD treatment, especially targeting calcium channels and synaptic regulation early in withdrawal, while acknowledging shared neuroadaptations in long-term treatment strategies to reduce craving and prevent relapse.

## Data Availability

The original contributions presented in the study are included in the article/supplementary material, further inquiries can be directed to the corresponding author.
